# Release properties of individual presynaptic boutons expressed during homosynaptic depression and heterosynaptic facilitation of the *Aplysia* sensorimotor synapse

**DOI:** 10.3389/fncel.2013.00165

**Published:** 2013-09-24

**Authors:** Guy Malkinson, Micha E. Spira

**Affiliations:** Department of Neurobiology, Institute of Life Sciences, The Hebrew University of JerusalemJerusalem, Israel

**Keywords:** homosynaptic depression, heterosynaptic facilitation, dishabituation, release sites, presynaptic boutons, calcium, synaptic potential, Aplysia

## Abstract

Much of what we know about the mechanisms underlying Homosynaptic Depression (HSD) and heterosynaptic facilitation is based on intracellular recordings of integrated postsynaptic potentials (PSPs). This methodological approach views the presynaptic apparatus as a single compartment rather than taking a more realistic representation reflecting the fact that it is made up of tens to hundreds of individual and independent Presynaptic Release Boutons (PRBs). Using cultured Aplysia sensorimotor synapses, we reexamined HSD and its dishabituation by imaging the release properties of individual PRBs. We find that the PRB population is heterogeneous and can be clustered into three groups: ~25% of the PRBs consistently release neurotransmitter throughout the entire habituation paradigm (35 stimuli, 0.05 Hz) and have a relatively high quantal content, 36% of the PRBs display intermittent failures only after the tenth stimulation, and 39% are low quantal-content PRBs that exhibit intermittent release failures from the onset of the habituation paradigm. 5HT-induced synaptic dishabituation by a single 5HT application was generated by the enhanced recovery of the quantal content of the habituated PRBs and did not involve the recruitment of new release boutons. The characterization of the PRB population as heterogeneous in terms of its temporal pattern of release-probability and quantal content provides new insights into the mechanisms underlying HSD and its dishabituation.

## Introduction

Homosynaptic depression (HSD or synaptic habituation) and its dishabituation by neuromodulators are conserved forms of learning and memory mechanisms at the synaptic level (Kandel, 2001; Fioravante and Regehr, 2011; Regehr, 2012). Experimental descriptions and analysis of these events are mainly based on intracellular recordings of excitatory or inhibitory postsynaptic potentials (PSPs) and the recordings of spontaneous miniature potentials. Because of the electro-anatomical properties of most neurons, intracellularly recorded PSPs are inevitably the integral of evoked neurotransmitter release from tens to hundreds of presynaptic boutons. Thus, conventional intracellular recordings do not permit analysis of the release behavior of individual presynaptic boutons during HSD and synaptic dishabituation.

Cultured *Aplysia* sensory-motor neurons (SN-L7) are extremely instrumental in unraveling molecular and cellular mechanisms that underlie different forms of short- or long- term synaptic plasticity (Byrne and Kandel, 1996; Bailey and Kandel, 2008; Glanzman, 2009; Gover and Abrams, 2009; Giachello et al., 2012; Wan et al., 2012). As in vertebrate CNS, the synaptic interactions between the SN and L7 are mediated by a large number of contact sites composed of Presynaptic Release Boutons (PRBs) that physically attach to the L7 mainly along the initial axonal segment (Malkinson and Spira, 2010b). In culture, the number of synaptic boutons formed between the SN and L7 is in the range of 30–40 (Kim et al., 2003; Malkinson and Spira, 2010a) and are spaced at 5–15 μm intervals (Malkinson and Spira, 2010a,b). Thus, PSPs recorded by an intracellular microelectrode represent an integral in time and space of an unknown number of individual active presynaptic boutons.

In cultured *Aplysia* neurons, HSD is generated by repetitive stimulation of the presynaptic neuron at low frequencies of 1–0.01 Hz (Byrne, 1982; Eliot et al., 1994). A number of hypotheses have been suggested to account for HSD (for a review see Gover and Abrams, 2009). At the presynaptic level HSD could reflect depletion of the available stores of transmitter (Gingrich and Byrne, 1985; Gingrich et al., 1988; Zhao and Klein, 2002, 2004), reduction in the probability of release by activity-dependent inactivation of the presynaptic voltage-gated calcium channels (Klein and Kandel, 1978), or by activity-dependent switching-off of molecular release mechanisms (Royer et al., 2000; Gover et al., 2002; Gover and Abrams, 2009; Wan et al., 2012). Postsynaptic mechanisms have also been considered, including receptor-inactivation by desensitization, but were ruled out (Armitage and Siegelbaum, 1998). The depressed synapse undergoes rapid facilitation (synaptic dishabituation) in response to a single bath application of 10 μM 5HT (Hochner et al., 1986a,b; and for review see Glanzman, 2008). Based on pharmacological experiments, it was suggested that the 5HT-induced synaptic dishabituation results from PKC activation (Braha et al., 1990; Ghirardi et al., 1992; Byrne and Kandel, 1996; Manseau et al., 2001), which in turn, induces the mobilization of neurotransmitter-containing vesicles from a non-releasable pool to the depleted pool of readily releasable vesicles (Gingrich and Byrne, 1985; Bailey and Chen, 1988; Klein, 1995; Byrne and Kandel, 1996; Zhao and Klein, 2002, 2004). Alternative explanations have also been considered namely, that PKC activates voltage-gated calcium channels strategically located close to the release site or that PKC act directly on the exocytotic machinery (Byrne and Kandel, 1996; Kandel, 2001; Zhao and Klein, 2002).

As intracellular recordings from the postsynaptic L7 neuron integrate in time and space the contribution of tens of presynaptic boutons, the interpretation of the results to the adapted models assumed that the presynaptic boutons operate as a homogeneous population, or actually as a single presynaptic terminal. However, this view fails to consider the structural heterogeneity of individual PRBs in terms of vesicle number and availability (Bailey and Kandel, 2008).

Here we used postsynaptic imaging of transient increases in the free intracellular calcium levels following evoked release in cultured SN-L7 synapses to examine the properties of individual PRBs during HSD and dishabituation. As we showed earlier, live-imaging of Excitatory Post-Synaptic Calcium concentration Transient (EPSCaT) by confocal imaging provides sufficient spatial and temporal resolution to resolve EPSCaTs generated by the release of neurotransmitter from individual PRBs (Malkinson and Spira, 2010b).

Our results reveal that the population of PRBs is heterogeneous, both in terms of the temporal pattern of release failures and their quantal content. This heterogeneous population can be clustered into three groups: boutons that consistently release throughout the habituation paradigm and are of high quantal content, boutons that intermittently fail to release transmitter immediately at the onset of the paradigm, (and also have low quantal content), and boutons that exhibit intermittent failure only after the tenth stimulation. 5HT-induced synaptic dishabituation is generated by the enhanced recovery to release transmitter by the habituated presynaptic boutons and does not involve the recruitment of new PRBs.

## Results

### Homosynaptic depression

**Figure 1:**
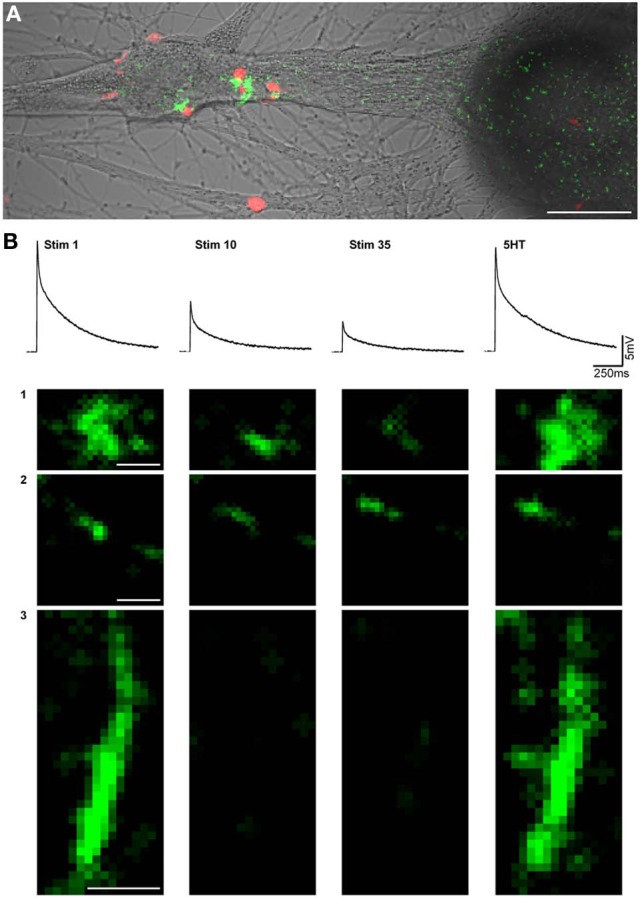
**Imaging of excitatory postsynaptic free calcium concentration transients (EPSCaT) during homosynaptic habituation and 5HT-induced dishabituation. (A)** Five days after plating, the SN was loaded with the calcium indicator rhod-2 (red), and the L7 was loaded with the calcium indicator fluo-4 (green). The image shown is an overlay of EPSCaTs (green) with apposed presynaptic boutons (red) on a transmitted image of the neurons. The image was processed for display purposes. **(B)** Sample EPSPS and high magnification images of corresponding EPSCaTs from a different experiment than **(A)**, monitored during a train of 35 stimuli delivered at 0.05 Hz, at three different sites (1, 2, 3). Shown are the 1st, 10th, and 35th stimuli, and the dishabituated signal, 5 min after bath application of 5 HT. In the example, the fluorescent intensity of the first EPSCaTs was set to a similar level. In sites 1 and 2 gradual reductions in the intensities were imaged. In site 3 the fluorescent signal disappeared on the 10th and 35th stimulus. 5HT application led to the recovery of the signals in all three sites. Scale bars in **A** = 20 μM. In **B** = 1 μM.

**Figure 2:**
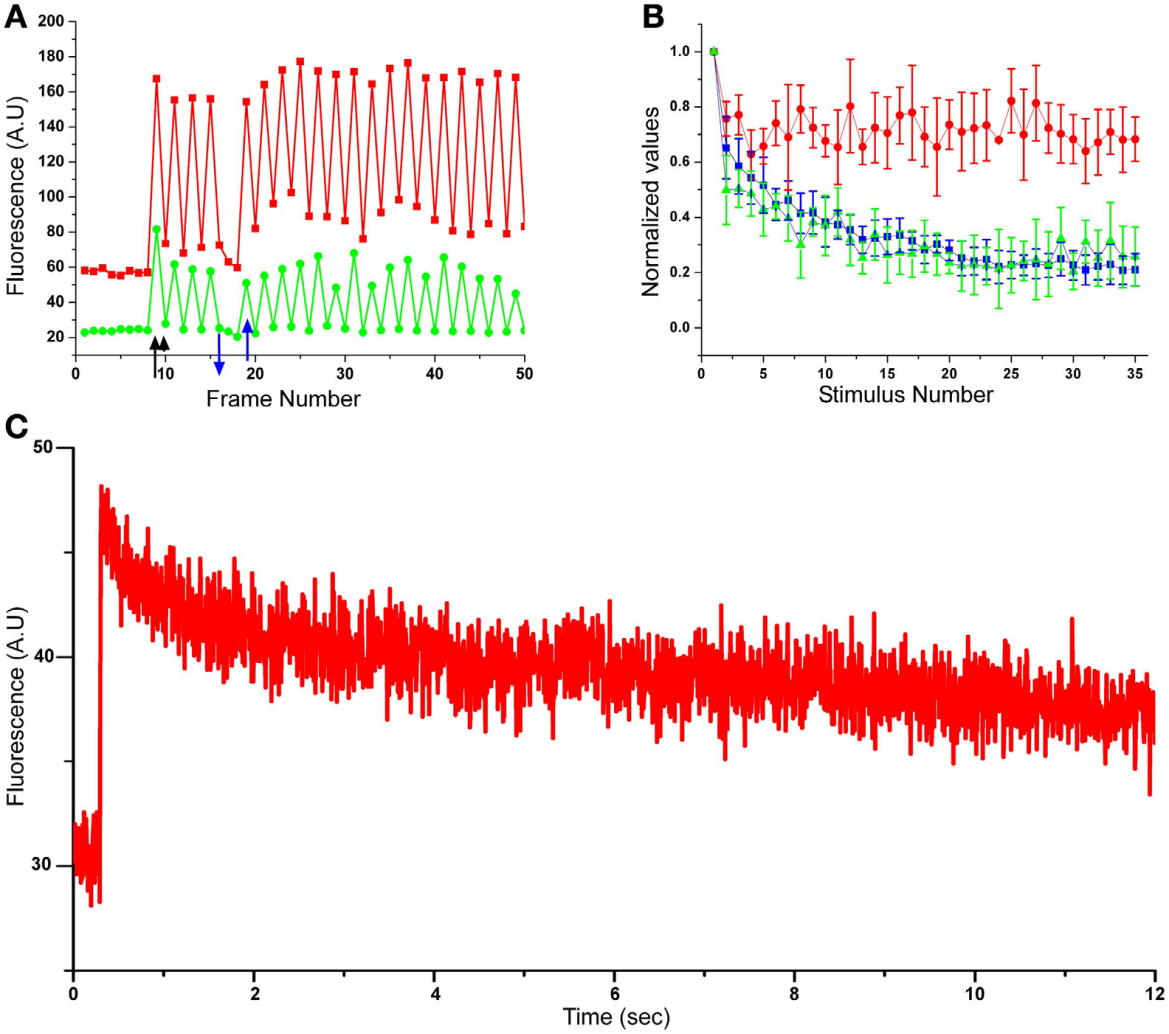
**Concomitant monitoring of EPSPs, pre- and postsynaptic calcium concentration transients during homosynaptic habituation. (A)** Raw data of pre- and postsynaptic calcium imaging from a single release site. In red, presynaptic rhod-2, in green the corresponding EPSCaT imaged by fluo-4 (green). A few images were grabbed from the presynaptic bouton and the corresponding postsynaptic site prior to presynaptic stimulation. The fluorescent signals generated by the first stimulus (indicated by an arrow) generated increased pre- and postsynaptic fluorescent signals. The next value on the graph (indicated by an arrowhead) was grabbed 10 s later when no stimulus had been delivered. Note that because of the long decay time of the presynaptic calcium concentration **(C)**, the baseline of the calcium concentration gradually increased in response to the consecutive individual stimuli but reached saturation, i.e., did not continue to increase. When the presynaptic stimuli were stopped (blue arrow pointing down) the rhod-2 signal recovered to the control level (this and the control stretch prior to stimulation demonstrate that no bleaching of the fluorescent signals are taking place). The gradual increase in the rhod-2 fluorescence is attributed to the slow relaxation time of the presynaptic calcium concentration transients **(C)**. The amplitudes of the EPSCaT (green) temporally correspond to the presynaptic rhod-2 signals and reveal changes in their maximal amplitude. It should be noted that when the stimuli delivered to the SN were stopped (blue down and up arrows), EPSCaTs were not recorded and the Δ*F*/*F*_0_ value recovered to control levels. **(B)** Pooled data from 6 experiments. The graphs show the average ± std of the recorded EPSPs (blue trace), EPSCaTs (green trace) and the presynaptic rhod-2 signals (red trace) during synaptic depression. Note that the normalized amplitudes of the EPSP and EPSCaT decline with time, whereas the presynaptic calcium does not. The rates of EPSPs and EPSCATs are depressed with similar time course and to a similar level. **(C)** Presynaptic calcium concentration transient kinetics as revealed by line-scan imaging through a single presynaptic bouton labeled with rhod-2. Note the slow decay time of the fluorescent signal.

To examine synaptic release by individual PRBs, we used electrophysiological recordings combined with optical monitoring of stimulus-evoked EPSCaTs by individual PRBs, as described previously (Malkinson and Spira, 2010b). The experiments were conducted on 4–5 day old cultured pairs of SN-L7 neurons (overall, *n* = 30 pairs). The calcium indicator rhod-2 or the inert dye Alexa555 were pressure injected into the SN, and fluo-4 was pressure-injected into the L7 using sharp intracellular glass micropipettes. At the end of the injection, the micropipettes were pulled out to avoid overloading the neurons with the indicators. After the dyes had equilibrated throughout the neurons, an extracellular electrode for stimulation of the SN and an intracellular microelectrode for recording from the L7 were inserted. Synaptic habituation was generated by the application of 35 presynaptic stimuli at 0.05Hz, and the postsynaptic responses were recorded while confocal-imaging the fluorescent signals generated pre- and postsynaptically (by rhod-2 and fluo-4, respectively) (Figure 1). The rate of confocal image grabbing was once every 10 s (twice that of the stimulation rate). This ensured that each stimulation frame had its own previous baseline frame (Figure 2A). To facilitate the imaging of the EPSCaTs, the experiments were performed in artificial sea water (ASW) in which the calcium concentration was 55 mM, instead of 11 mM and the magnesium concentration was reduced to 11 mM. Consistent with earlier studies that reported that this concentration of divalent ions does not change synaptic strength (Jiang and Abrams, 1998; Royer et al., 2000), we found that the SN-L7 synapses underwent HSD in high Ca-ASW with the same kinetics as in normal ASW.

The results were analyzed off-line. For the analysis we selected EPSCaTs with discernible apposed presynaptic varicosities using the most intense EPSCaTs images, normally generated by the first stimulus, corresponding to the first EPSP. The selected regions exhibiting EPSCaTs are referred to as regions of interest (ROIs) and represent a functional PRB.

We analyzed the integrated fluorescence intensity of individual EPSCaTs as a function of the consecutive number of stimuli within the selected ROI. With successive stimuli, the fluorescence intensity in different ROIs either diminished completely or partially (Figure 1B). To establish the quantitative relationships between the recorded EPSCaT and the intracellularly recorded EPSP, we analyzed the electrophysiological and fluorescence data as follows (*n* = 6 pairs): the normalized EPSP values (with respect to the first EPSP) were calculated and plotted to generate an average, normalized EPSP habituation kinetics curve (Figure 2B, blue trace). The average values of the presynaptic Δ*F*/*F*_0_ Rhod-2 (Figure 2B, red) and postsynaptic Fluo-4 EPSCaTs (Figure 2B, green) fluorescent values at each ROI were calculated. These averaged EPSCaT were normalized in respect to the fluorescent intensity generated by the first stimulation and plotted as a function of the consecutive stimuli (time) in a similar manner to that of the averaged EPSPs amplitudes. (Figure 2B, red and green traces).

Examination of the decline kinetics of the average EPSPs and the average integrated EPSCaT showed that they were very similar. Thus, whereas the first EPSP underwent HSD to an average of 21 ± 4% (avg ± standard deviation, std) of its initial amplitude within 35 stimuli, the integral of the EPSCaTs diminished to an average level of 19 ± 9% of their initial average value (Figure 2B).

To determine how the behavior of the EPSCaT profile depicts the EPSP kinetics we cross-correlated between the EPSCaT profile and the EPSP profile in each experiment (*n* = 10 pairs) to obtain their *r* and *p*-values. The mean *r*-value was 0.84 ± 0.08 (average ± std, *p* < 0.001). This result, together with our previously published data (Malkinson and Spira, 2010b) suggests that the integral of the EPSCaT fluorescence correlates well with the electrically recorded PSPs and that therefore it is reasonable to assume that the EPSCaTs are also proportional to the number of quanta (q.c.) released from the population of active presynaptic boutons.

HSD as revealed by measuring the EPSP amplitude or EPSCaT could be the outcome of reduced calcium influx to the SN presynaptic terminals. Technically it is impossible to measure the calcium concentration transient right at the neurotransmitter release sites. Nevertheless, measurements of the spatiotemporal calcium transient imaged from SN neurites may shed some light on the question. Consistent with earlier studies (Armitage and Siegelbaum, 1998), concomitant recordings of the transient elevations of the [Ca^2+^]_*i*_ from the presynaptic neuron and the postsynaptic cell (with rhod-2 and fluo-4, respectively, Figure 2B) revealed that the average transient elevation of the [Ca^2+^]_*i*_ in the presynaptic SN during the HSD did not decrease with successive stimuli. It should be noted that a ~20% drop in the presynaptic Δ*F*/*F*_0_ transient [Ca^2+^]_*i*_ values was estimated for the second stimulus (in respect to the first one, Figure 2B). This large drop is the outcome of the method used to estimate the Δ*F*/*F*_0_ fluorescent intensity values. Thus, the first Δ*F*/*F*_0_ value was obtained by dividing the amplitude of the first EPSCaT by the control fluorescent value of the rested neuron (Figure 2A red trace). The second Δ*F*/*F*_0_ value was obtained by dividing the peak amplitude value of the second EPSCaT by the preceding fluorescent value generated during the recovery time of the former (first) EPSCaT. Since the recovery time of an EPSCaT is longer than the time interval between stimuli, the calculated Δ*F*/*F*_0_ is smaller. Whereas the recovery time of the calcium-indicator fluorescent signal following a single stimulation to the presynaptic SN is close to 30 s, the time interval between the stimuli applied to the presynaptic neuron is 20 s (Figure 2C). As a consequence of the above, the Δ*F*/*F*_0_ value of the second stimulus is an underestimate of the first one. To further demonstrate this point, in the experiment shown in Figure 2A we stopped stimulating the presynaptic neuron (up and down blue arrows). This indeed allowed for the relaxation of the presynaptic calcium level to the control value within only ~40 s. Interestingly, consecutive stimuli delivered to the SN (after the first stimuli) did not lead to a considerable buildup of rhod-2 fluorescent signal level and therefore the *F*_0_ maintained similar levels (Figure 2A). The mechanism(s) that sets the free intracellular calcium level at a rather constant level is not known but was observed in earlier studies (for example Blumenfeld et al., 1990).

### Analysis of the release properties of single presynaptic boutons

After establishing that the average kinetics of the integrated EPSCaT intensity undergoes a parallel depression to the habituation of the EPSP, we proceeded to characterize the behavior of individual PRBs as reflected by the fluorescent signal intensity of individual EPSCaTs. To that end, we examined 235 individual PRBs from 10 pairs. As noted, the decline in the summed EPSCaT fluorescence (Figure 2B) is the outcome of either a decline in the number of functional PRBs or a decline in the amount of neurotransmitter released from individual boutons (manifested as a reduction in the EPSCaT intensity), or a combination of both. We therefore monitored and calculated the fraction of presynaptic stimuli that evoked individual EPSCaTs (i.e., the number of times an EPSCaT was generated by a given ROI, divided by the total number of stimuli, which yielded the bouton release probability). A positive evoked release event was defined when the EPSCaT exceeded a 5% confidence interval that was calculated individually for each ROI (see Materials and Methods).

**Figure 3:**
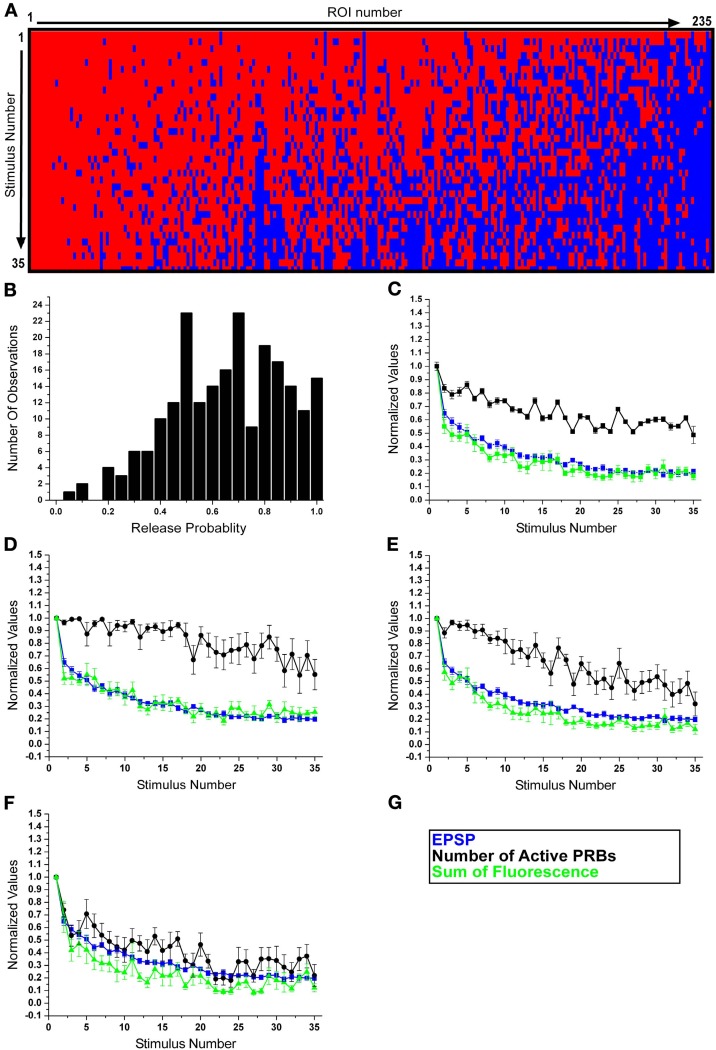
**Subdivision of the release boutons into three phenotypes. (A)** A raster plot of all EPSCaTs (corresponding to 235 release boutons documented in this study). Red depicts a release event, blue failure of release to occur. Boutons revealing consistent release over the 35 stimuli are depicted on the left hand side of the graph, and those that revealed failures to release within the first 10 stimuli are grouped on the right side of the graph. **(B)** The histogram depicts the normalized EPSCaT Δ*F*/*F* values of 235 PRBs over 35 presynaptic stimuli at 0.05 Hz. Note the peaks around 0.5, 0.7, and 0.80. **(C–F)** Normalized data from 10 experiments. In blue the normalized EPSP amplitudes, in green the normalized sum of EPSCaTs of the PRBs and in black the normalized number of active PRBs as a function of the stimulus number. **(C)** Note that the overall average decline kinetics of the EPSPs amplitude and all EPSCaTs reveal similar kinetics. After 35 stimuli both EPSPs and EPSCaT decline to about 20% of the initial level. HSD is associated with a smaller 40% decline in the number of active PRBs. **(D–F)** The population of release boutons was subdivided into three release groups. Shown in **(D)** is group A, which represents the behavior of 25% of the release PRBs studied. The gradual decline of the EPSCaT develops in parallel to that of the EPSPs but the number of release boutons does not decline over the 35 stimuli. This result suggests that in this group the decline in the EPSCaT is mainly the outcome of reduced quantal content. Shown in **(E)** is group B, which represents the behavior of 36% of the PRBs studied. The depression of the EPSP and EPSCaT is associated with ~40% decline in the number of the PRBs. Shown in **(F)** is group C, which represents the behavior of 39% of the PRBs studied. The variability in the number of the release sites is very large (Data in **D–F** are presented as average ± sem).

Examination of the release behavior of the individual boutons revealed that the release probability of an individual PRB ranged from 0.05 to 1 (Figures 3A,B). Shapiro–Wilk test indicated that the data is not normally distributed. A tri-modal mixture analysis was applied to detect the possible sub-distributions (of the release probability) which form the overall distribution. Qualitatively, the population of PRBs could be subdivided into three groups, and the tri-model analysis is consistent with the above three subgroups. We can thus tentatively suggest that the PRBs can be viewed as follows: (A) A group that presented almost no failures during the 35 stimuli leading to HSD (left hand side of Figure 3A), (B) A group that initially did not display failures but with time the number of failures increased (the central part of Figure 3A), (C) A group that exhibited release failures from the very beginning of the train of stimuli and throughout its entire duration (right hand side of Figure 3A).

To get a quantitative sense of the parameters that differentiate these 3 groups, we compared the release behavior of each group along different time frames of the HSD paradigm. To that end, the HSD paradigm was divided into 3 temporal frames: stimuli 1–10, stimuli 11–20, and stimuli 21–30. For each group the average number of successfully evoked release events was calculated. The data indicated that group A had 9.5 ± 0.7 (avg ± std) successes for stimuli 1–10 to evoke release, 8.9 ± 0.9 successes for stimuli 11–20 and 9.5 ± 1.4 successes for stimuli 21–30. The release successes of group B in the same time frames were 8.7 ± 1.4, 7.3 ± 2, and 5.5 ± 1.9, and the successes of group C were 5.5 ± 2.3, 4 ± 2.3, and 2.9 ± 1.9. We tested for significant differences among the groups in each frame with One-Way ANOVA, which showed a significant difference between the groups [frame 1–10: *F*_(2, 234)_ = 125.032, *p* < 0.001, frame 11–20: *F*_(2, 234)_ = 128.252, *p* < 0.001 and frame 21–30: *F*_(2, 234)_ = 216.241, *p* < 0.001]. We then performed Fisher LSD *post-hoc* tests between pairs of each group, and found that all pairwise comparisons were significant. These results demonstrate that differences in release probability between the groups are already apparent in the first time frame.

Using these quantitative criteria the population of the PRBs was characterized as follows (Figures 3A,B): group A—comprising 25.3 ± 13% (avg ± std) of the population (*n* = 235 PRBs) was characterized by an overall release probability of 0.80–1. Group B comprised 35.9 ± 16.4% of the population and had a release probability of 0.6–0.75 and group C comprised 38.7 ± 18.7% of the population and exhibited a 0.05–0.55 probability to release.

The subdivision of the PRBs according to the release probability also corresponds to the initial fluorescent intensity of the EPSCaT signal. One Way ANOVAs showed a significant difference between the average Δ*F*/*F*_0_ values of the first EPSCaTs of the three groups (0.54 ± 0.06, 0.28 ± 0.02, and 0.2 ± 0.02, respectively (average ± sem) *F*_(2,232)_ = 9.511, *p* < 0.001). *Post-hoc* Fisher LSD tests between pairs of the groups showed significant differences for all pairs. More specifically, in group A, the initial signal was the highest and gradually declined during habituation (Figure 3D green). The behavior of these boutons was consistent with PRBs with a large number of readily releasable vesicles, in which the number of released quanta gradually declines during habituation but is not depleted. Group B revealed a gradual decline in the EPSCaT fluorescent intensity (Figure 3E green) and in addition also showed intermittent release failures. The behavior of these boutons was consistent with PRBs with a smaller number of readily releasable vesicle pool or functional release sites within a single PRB. Thus, after a number of stimuli, the vesicle pool in individual PRBs is exhausted and intermittent failures to release are monitored. Group C was characterized by a significant drop in the number of releasing boutons immediately following the first stimulus (Figure 3F); concomitantly the fluorescent intensity of the EPSCaT was reduced. In fact, after 10–15 stimuli, the summed contribution of the presynaptic boutons (of this pool) to the EPSCaT fluorescence was nearly 30% of the initial value. This behavior could represent presynaptic boutons whose release-competent pool of vesicles is very small or indicate that the number of release sites within a single PRB is small and thus intermittent failures to release are observed immediately after the first stimulus.

Examination of the pooled data revealed that the reduction in the number of active PRBs contributed less than the reduction in the quantal content of the active boutons. The levels of contributions of these parameters to habituation were estimated to be ~50% (100–50%, Figure 3C, black trace) of the reduction in the number of functional of PRBs and ~80% (100–20%, Figure 3C, green trace) of the reduction in the quantal content (see also Table 1, column A, rows 1 and 3).

**Table 1:** **The changes in the number of the PRBs (rows 1 and 2) and the strength of neurotransmitter released from the PRBs (rows 3 and 4) during HSD (rows 1 and 3) and 5HT induced dishabituation (rows 2 and 4)**.

		**A**	**B**	**C**	**D**
		**All groups**	**High (Group A)**	**Med (Group B)**	**Low (Group C)**
1	Average ratio between **number** of EPSCaTs in the last habituation stimulus to their **number** in the 1st habituation stimulus	0.48 ± 0.06	0.55 ± 0.12	0.32 ± 0.09	0.22 ± 0.08
2	Average ratio between **number** of EPSCaTs in the dishabituation stimulus to their **number** in the 1st habituation stimulus	0.9 ± 0.04	0.95 ± 0.03	0.9 ± 0.03	0.84 ± 0.06
3	Average ratio between the **sum** of fluorescence of EPSCaTs in the last habituation stimulus to their **sum** in the 1st habituation stimulus	0.18 ± 0.02	0.25 ± 0.03	0.12 ± 0.04	0.12 ± 0.03
4	Average ratio between the **sum** of fluorescence of EPSCaTs in the dishabituation stimulus to their **sum** in the 1st habituation stimulus	0.67 ± 0.05	0.61 ± 0.05	0.7 ± 0.19	0.66 ± 0.07

The columns provide the values of the 3 groups of PRBs (columns B, C, D) and the pooled data for all PRB types (column A). Data is presented as (avg ± sem).

The relative sum of fluorescent intensity generated by the EPSCaTs of PRB groups A, B, and C in the last stimulus of the habituation was 0.25 ± 0.03 (avg ± sem), 0.12 ± 0.04, and 0.12 ± 0.03 with respect to the relative sum of the fluorescence in the first stimulus (Table 1, row 3, columns 2–4). In other words, in the last stimulus, the quantal content that was released was still ~2 times higher in group A than in group C, similar to the 1st EPSCaT.

### Dishabituation (heterosynaptic facilitation)

Next we examined the mechanisms underlying short-term *5HT*-induced heterosynaptic facilitation (Brunelli et al., 1976; Castellucci and Kandel, 1976; Abrams et al., 1984; Gover and Abrams, 2009).

As established previously, a single bath application of 5HT to a homosynaptically depressed synapse leads to synaptic dishabituation. In the present study, 5 min after the 5HT application, the EPSP amplitude recovered from 21 ± 1% (avg ± sem) to 75 ± 7% with respect to the first stimulus and the EPSCaT signal recovered from 21 ± 2% to 67 ± 5% (with respect to the first stimulus). On average, per experiment, the ratio of EPSCaT recovery to the EPSP recovery was 90 ± 19%. Theoretically, 5HT-induced synaptic dishabituation could be due to recovery of the release properties of individual PRBs or the recruitment of new PRBs. To differentiate between these possibilities we analyzed the relative contribution of the three PRB groups before and after 5HT application (Table 1). We found that (A) the total number of PRBs that appeared in the last stimulus of the HSD paradigm dropped to 48 ± 6% (avg ± sem), the 5HT application increased the number of PRBs to 90 ± 4% of that counted after the first stimulus, (B) the total sum of EPSCaT fluorescence generated by all 3 groups in the last stimulus of the HSD paradigm was 18 ± 2% with respect to the first stimulus (Figure 3C). This signal recovered to 67 ± 6% (Table 1).

The relative EPSCaT sum of fluorescent intensity generated by PRB groups A, B, and C in the last stimulus of the synaptic habituation was 0.25 ± 0.03 (avg ± sem), 0.12 ± 0.04, and 0.12 ± 0.03 with respect to the total EPSCaT fluorescence in the first stimulus. After application of 5HT, the summed EPSCaTs of groups A, B, C reached to 0.61 ± 0.05, 0.7 ± 0.19, and 0.66 ± 0.07 of that generated by the first stimulus of rested synapses. These data suggest that 5HT-induced synaptic dishabituation was generated by a ~2-fold increase in the number of active PRBs with respect to their number at the end of the habituating paradigm, and an overall ~3-fold increase of the quantal content. This is subdivided as follows into the three release groups: in group A, in which the number of PRBs was only slightly reduced, the recovery in terms of number was 95 ± 3% (avg ± sem) and the increase in EPSCaT ranged from 25 ± 3 to 61 ± 5%. In group B the recovery in terms of number was 90 ± 3% and the increase in EPSCaT ranged from 12 ± 4 to 70 ± 19%, and in group C the recovery in terms of number was 84 ± 6% and the increase in EPSCaT ranged from 18 ± 3 to 66 ± 7% (see Table 1, columns 2–4). Since the imaging revealed that the 5HT-induced facilitation was associated with increased postsynaptic intensity fluorescence across the control PRBs sites and was not associated with the appearance of new active PRBs, a single 5HT application most likely leads to the recovery of the original population of PRBs, in comparison to the last stimulus of the habituation, rather than the recruitment of new PRBs.

## Discussion

The present study examines the process of HSD and 5HT-induced synaptic dishabituation for the first time at the level of individual release boutons that comprise the classical sensory motor synapse from the gill withdraw reflex of Aplysia. Simultaneous confocal imaging of EPSCaTs evoked by presynaptic stimulation revealed that the population of PRBs has heterogeneous release properties. To simplify the description of this heterogeneous population and based on the temporal pattern of release failures by individual PRBs (and statistical assessments) we subdivided the PRB population into three groups: (A) A group that reveals almost no failures during the 35 stimuli, (B) A group that initially does not exhibit failures but with time the number of failures increases. (C) A group that exhibits release failures from the very beginning of the train of stimuli and throughout its entire duration.

Group A, comprising 25% of the functional presynaptic boutons, was characterized by the largest initial EPSCaT signals. These EPSCaTs generated ~35% of the grand total of EPSCaT generated by all release boutons during the first EPSP, and ~60% of the integrated EPSCaT signal when the EPSP was depressed to 20% of its initial value. These release properties of phenotype A presynaptic boutons are consistent with a relatively large store of releasable vesicles which is replenished with a fast time constant with respect with to the rates of its release during HSD or/and in combination with a large number of release sites which are gradually turned off or remain operative throughout the HSD paradigm.

Group B comprised 36% of the presynaptic boutons which began to drop out only after the first 10 stimuli. This group contributed 39% of the EPSCaT signal generated by the first EPSP and only ~24% of the signal generated by the EPSCaT at the end of the HSD paradigm. This presynaptic cluster behaved as though it consisted of presynaptic boutons in which the releasable vesicle store was limited and where the replenishment of the store was insufficient to maintain release after its partial depletion. Alternatively, this group could represent PRBs which harbor a number of release sites and that these are turned off only after ~10 stimuli.

Group C comprised 39% of the functional presynaptic boutons and was characterized by a very small releasable pool that began to show intermittent events of successful releas**e** immediately after the first stimulus. It is also conceivable that this group of PRBs harbors a single release-site bouton which is turned off or on intermittently. This phenotype contributed only 26% of the integrated EPSCaT of the first EPSP and ~12% during the depressed state of the EPSP. It should be noted that we cannot discount an alternative explanation for the behavior of phenotype C which assumes that the fluorescent intensity of the EPSCaT in C was close to the threshold level of EPSCaT detection used in this study. This low signal could reflect the distance of the synapse from the focal plane of the confocal microscope. Thus, a small reduction in the EPSCaT signal could have been counted as failure to release. Although group C comprised 39% of the functional presynaptic boutons, its relative contribution to the EPSCaT signal was only 25% of the initial EPSCaT. Thus, even if it represents a methodological artifact, immature release boutons, or innate properties related to its location in respect to the cell body, its contribution was small.

Heterosynaptic facilitation mimicked by the application of 5HT to the bathing solution led to the recovery of the presynaptic bouton phenotypes to their original level of representation. That is, groups A, B, and C comprised the same fraction of active boutons before HSD and after dishabituation. This is consistent with findings in the literature that one application of 5HT alters the release level by covalent modification of existing target proteins distributed throughout the neuronal structures. These include phosphorylation of potassium channels that lead to increased calcium influx into the boutons (Castellucci et al., 1980, 1982), the enhanced mobilization and supply of release competent vesicles (Humeau et al., 2001; Khoutorsky and Spira, 2008, 2009) or possibly the activation of silenced release sites (Gover and Abrams, 2009). The single 5HT application did not alter the preexisting representation levels of the different presynaptic boutons phenotype, and did not recruit silent release boutons. This suggests that 5HT-induced short-term synaptic dishabituation upregulates preexisting common mechanisms in all three presynaptic bouton phenotypes.

### Which mechanisms underlie the heterogeneous release properties of presynaptic boutons?

Differences in the release probability of presynaptic boutons generated by a single neuron can be attributed to a number of cell biological variables. These include differences in the distribution and densities of voltage-gated calcium channels (Sheng et al., 2012), local differences in the calcium buffering capacities of the boutons (Zhang and Linden, 2009, 2012), the availability of releasable vesicles (Holderith et al., 2012) the rate at which the released pool recovers (Sankaranarayanan and Ryan, 2000), and local variability in the molecular complexes that interface vesicles with the plasma membrane and sense the incoming calcium ions (Zhang and Linden, 2009, 2012, for review see Branco and Staras, 2009).

Currently our EPSCaT imaging method does not allow us to document miniature potentials or neurotransmitter release from individual release sites within a single PRB; nor does it have the sensitivity to enable the analysis of the quantal content of evoked release by calculating the standard deviations of the EPSCaT. Nevertheless, it is reasonable to assume that the qualitative differences in the EPSCaT fluorescence intensities correspond to differences in the evoked release properties of the individual presynaptic boutons (Bailey and Chen, 1983, 1988).

The sensory motor neuron synapse is typically schematically depicted in the literature as an axon that branches out parallel multiple presynaptic elements. This schema places all presynaptic terminals at equal distance from the cell body, hence implying equal probabilities for the distribution of subcellular organelles, proteins and mRNA (Kim et al., 2003; Bailey and Kandel, 2008). Whereas significant differences in the properties of the individual terminal boutons are considered in relation to long-term plasticity of the neurons, the underlying mechanisms are not discussed (Kim et al., 2003). Intuitively if all terminal boutons are equally distributed from the cell body, no innate presynaptic differences in their subcellular structure and physiology would be expected.

In reality, the presynaptic boutons of the sensory neurons as well as those of vertebrate neurons are spaced sequentially along relatively long neurites (Malkinson and Spira, 2010a). Thus, the probability that proximal boutons will be supplied by fresh Golgi-derived vesicles, mitochondria, proteins and RNA is greater than the probability for remote presynaptic boutons. It is therefore conceivable that because of internal gradients, the properties of presynaptic boutons may differ. Thus, the unequal distribution of organelles and molecules along a single neurite could account for the findings presented here (Fisher-Lavie et al., 2011).

In interpreting the results it is important to recall that to enhance the EPSCaT fluorescent signals we conducted our experiments in high-calcium/low-magnesium ASW. Such conditions slightly increase the driving force for calcium influx, and the effective permeability of calcium channels, since competition with magnesium ions is reduced. Although these conditions may theoretically alter the behavior of the PRBs it still enabled us to document for the first time that the PRBs formed by a single presynaptic neuron on a single postsynaptic cell are composed of a heterogeneous population. The relative composition of the heterogeneous population was not altered after synaptic facilitation by 5HT. Since our confocal imaging restricts the observations to a relative narrow focal plane and since this focal plane was maintained it is safe to assume that the same population of PRBs was imaged throughout a single experiment. Nevertheless, we expect that as improved calcium probes are developed, our approach to studying the properties of individual PRBs by monitoring EPSCaTs will enable researchers to run experiments with higher fidelity and at lower calcium levels.

In summary, our observations suggest that the typical kinetics of HSD of the SN-L7 synapse are the outcome of the heterogeneous release properties of populations of presynaptic boutons. Short term synaptic dishabituation by 5HT is generated by the facilitated replenishment of the depleted vesicle stores or the recovery of inactivated release machinery and does not involve the recruitment of new silent presynaptic boutons.

## Methods

### Cell culture

Sensory (SN) and L7 motoneurons (MN) were isolated from pleural and abdominal ganglia of Aplysia californica (imported from the Marine resources facility at the University of Miami, FL, USA) and maintained in culture as previously described (Schacher and Proshansky, 1983; Spira et al., 1996).

### Culture medium

The culture medium consisted of 10% filtered hemolymph obtained from Aplysia fasciata collected along the Mediterranean coast, diluted in modified Leibovitz's L-15 medium (Spira et al., 1996). ASW consisted of 460 mM NaCl, 11 mM KCl, 55 mM CaCl2, 10 mM MgCl2, and 10 mM Hepes adjusted to pH 7.6.

### Chemicals and pharmacological reagents

Alexa-555 (Invitrogen), Rhod-2 (Teflabs) and Fluo-4 (Invitrogen) were prepared as 10 mM stock solutions in 0.5M KCl. 5HT was prepared as a 10 mM stock solution in DDW and added to the bath to a final concentration of 10 μM.

### Confocal microscope imaging

The systems used for confocal imaging consisted of a Nikon C1 confocal system mounted on a Nikon TE-2000 Eclipse microscope system with a Nikon plan-Apo chromat 60× 1.4 NA oil objective or plan-fluo 40× objective. This system is equipped with 3 lasers: blue diode (405 nm), Argon (488 nm) and Green HeNe (543 nm). Images were collected and processed using EZ-C1 software. This system was used for confocal imaging of all dyes. Fluo-4 was excited with the 488 nm laser and the emission was collected with 515/30. Alexa-555 and Rhod-2 were excited with the 543 nm laser, and the emission was collected with 605/75 nm.

Evoked alterations of EPSCaT were documented as described by us earlier (Malkinson and Spira, 2010b) either by using the line-scan mode along a 15–35 μm-long line at frequency of 5.9 ms per line or by laser scanning a region of interest (ROI) of varying dimensions with a pixel dwell-time of 7.68 μs. Under the experimental setting used in the described experiments and for the duration of each imaging of habituation or/and dishabituation stretches we did not observe bleaching of the fluorescent probes.

### Image analysis

Images were analyzed off-line using NIH ImageJ software (Bethesda, MD, USA) by manually selecting individual ROIs around points of interest. Only ROIs that exhibited fluorescent increases that exceeded a 5% confidence interval (see below) were chosen for further analysis. A kernel size of 0.4 was used to perform mean filtering (ImageJ) on some images for display purposes. The figures were prepared using Macromedia FreeHand software.

Calculation of 5% confidence interval: A custom-written Matlab (Mathworks) script was written. The script performed the following: the raw datasets were divided into one dataset (baseline dataset) that contained the images acquired before the stimulations, and another dataset (stimulation dataset) that contained the images acquired during the stimulations. Each dataset thus contained the same amount of data points. In order to assess the behavior of the baseline, the difference between each pair of consecutive baseline points was calculated, and the average (${\bar{\text{X}}}_{\text{differences}}$) and standard deviation of these differences (${\text{S}}_{\text{differences}}$) was calculated. The upper confidence interval was then calculated as: ${\bar{\text{X}}}_{\text{differences}}+\frac{S_{\text{differences}}}{\sqrt{n}}\times t_{\left(n-1\right)}$, with a confidence level of α = 0.05.

### Statistical analyses

ANOVA and *post-hoc* Fisher LSD tests were calculated with SPSS (IBM Corp. Released 2010. IBM SPSS Statistics for Windows, Version 19.0. Armonk, NY: IBM Corp.). Shapiro–Wilk test was performed with JMP, v9. EXCEL (Microsoft) was used for all other calculations.

### Electrophysiology

For stimulation and recording from the motoneurons, glass electrodes were pulled to resistances of ~7–10 MΩ, and filled with 2M KCl solution. Presynaptic stimulation was carried out by an extracellular electrode as described (Khoutorsky and Spira, 2005, 2009).

### Conflict of interest statement

The authors declare that the research was conducted in the absence of any commercial or financial relationships that could be construed as a potential conflict of interest.
